# Increased ratio of anti-apoptotic to pro-apoptotic Bcl2 gene-family members in lithium-responders one month after treatment initiation

**DOI:** 10.1186/2045-5380-2-15

**Published:** 2012-09-12

**Authors:** Lori Lowthert, Janine Leffert, Aiping Lin, Sheila Umlauf, Kathleen Maloney, Anjana Muralidharan, Boris Lorberg, Shrikant Mane, Hongyu Zhao, Rajita Sinha, Zubin Bhagwagar, Robert Beech

**Affiliations:** 1 Department of Psychiatry, New Haven, CT, 06511, USA; 2 Keck Foundation Biotechnology Biostatistics Resource, New Haven, CT, 06511, USA; 3 Center for Genome Analysis, New Haven, CT, 06511, USA; 4 Department of Epidemiology and Public Health, Yale University School of Medicine, New Haven, CT, 06511, USA; 5 Neuroscience Global Clinical Research, Bristol-Myers Squibb, Wallingford, CT, 06492-1996, USA

**Keywords:** Bipolar disorder, Microarray, Lithium-response, Gene expression, BCL2, Apoptosis, Mitochondria

## Abstract

**Background:**

Lithium is considered by many as the gold standard medication in the management of bipolar disorder (BD). However, the clinical response to lithium is heterogeneous, and the molecular basis for this difference in response is unknown. In the present study, we sought to determine how the peripheral blood gene expression profiles of patients with bipolar disorder (BD) changed over time following intitiation of treatment with lithium, and whether differences in those profiles over time were related to the clinical response.

**Methods:**

Illumina Sentrix Beadchip (Human-6v2) microarrays containing > 48,000 transcript probes were used to measure levels of expression of gene-expression in peripheral blood from 20 depressed subjects with BD prior to and every two weeks during 8 weeks of open-label treatment with lithium.

Changes in gene-expression were compared between treatment responders (defined as a decrease in the Hamilton Depression Rating Scale of 50% or more) and non-responders. Pathway analysis was conducted using GeneGO Metacore software.

**Results:**

127 genes showed a differential response in responders vs. non-responders. Pathway analysis showed that regulation of apoptosis was the most significantly affected pathway among these genes. Closer examination of the time-course of changes among BCL2 related genes showed that in lithium-responders, one month after starting treatment with lithium, several anti-apoptotic genes including Bcl2 and insulin receptor substrate 2 (IRS2) were up-regulated, while pro-apoptotic genes, including BCL2-antagonist/killer 1 (BAK1) and BCL2-associated agonist of cell death (BAD), were down-regulated. In contrast, in lithium non-responders, BCL2 and IRS2 were down-regulated, while BAK1 and BAD up-regulated at the one-month time-point.

**Conclusions:**

These results suggest that differential changes in the balance of pro- and anti- apoptotic gene-expression following treatment with lithium may explain some of the heterogeneity in clinical response in BD patients.

## Background

Bipolar disorder (BD) is a devastating neurobiological illness, affecting from 0.8% to 1.2% of the population [1-3]. Clinically, the disorder is characterized by episodes of mania and major depression. However, as with the vast majority of psychiatric illnesses, the nature of the underlying pathophysiology remains poorly understood [4]. Lithium is considered by many as the gold standard medication in the management of bipolar disorder (BD). It was the first treatment with demonstrated efficacy in BD [5] and it is still considered first line treatment for acute mania, acute bipolar depression, and maintenance treatment [6-8]. It is also the only medication to be consistently associated with a reduction in suicidal ideation or attempts in patients with BD [9-11].

Previous studies have investigated polymorphisms in a variety of genes including serotonin transporter, glycogen synthase kinase-3beta, inositol polyphosphatase 1-phosphate, brain-derived neurotrophic factor and activator protein 2beta, and found that these variants are not predictive factors for response to lithium [12,13]. Genome-wide association studies have also been conducted to identify common gene variants that may be associated with lithium response [14,15]. While some loci with suggestive evidence for linkage have been found [14], to date no SNPs have met the threshold for genome-wide significance. Thus, despite decades of work related to this topic, the molecular basis for the heterogeneity in response to treatment with lithium remains unknown [16].

Previous studies have shown that chronic treatment of animals [17,18] with lithium increases expression of the anti-apoptotic gene BCL2 and decreases the expression of the pro-apoptotic gene BAX. Treatment with lithium has also been shown to block the reduction of the BCL2/BAX ratio in animals treated with methamphetamine [19]. These findings have led to the suggestion that changes in the expression of BCL2 and related genes may be responsible for some or all of the therapeutic effects of lithium [17,20,21]. However, with current technologies it is impossible to assess the time-course of such changes in the brains of living subjects. Thus, whether such changes occur in human patients treated with lithium, and, if so, how they are related to differences in treatment outcome among patients remains unknown.

In a recently completed study conducted by our laboratory [22], we compared gene-expression profiles in whole blood of depressed subjects with BD to that of healthy controls. We identified a large number of genes whose expression was altered in the blood of depressed BD subjects. Strikingly, in that study, all of the top 10 functional pathways identified were interconnected, and related directly or indirectly to mitochondrial functions including energy metabolism and the regulation of apoptosis by mitochondrial proteins. One advantage of studying peripheral markers, including peripheral blood gene-expression, is that they can be assessed repeatedly over time, and thus compared directly to changes in clinical status. In the present study, we sought to determine how the peripheral blood gene expression profiles of patients with BD changed over time when they were treated with the mood stabilizer lithium, and whether differences in those profiles over time were related to the clinical response to lithium.

## Methods

### Subjects

Subjects included in this study are identical to those included in our previous publication [22]. All procedures involving human subjects were approved by the Yale Human Investigation Committee and are in accordance with the Helsinki declaration of 1975. All subjects provided written informed consent at the time of enrollment in the study. Inclusion criteria for subjects with BD included age between 18 and 65 years, diagnosis of bipolar disorder (bipolar type I or II), currently depressed as defined by Diagnostic and Statistical Manual of Mental Disorders, fourth edition text revision (DSM-IV- TR) [23], and not being treated with lithium at the time of study entry. Diagnosis was determined by consensus of clinical interview by a Board Certified Psychiatrist (RDB or ZB) and the Structured Clinical Interview for DSM-IV Axis I Disorders [24] (performed by KM, AM, or BL). Exclusion criteria included DSM-IV-TR diagnoses other than bipolar I or II, current or recent (past 30 days) abuse of illicit substances (verified by urine toxicology screening), pre-existing thyroid pathology (e.g. hypothyroidism or hyperthyroidism) as evidenced by an abnormal thyroid function test at screening, or history or evidence of a medical condition that would expose them to an undue risk of a significant adverse event or interfere with assessments of safety or efficacy during the course of the trial, including but not limited to hepatic, renal, respiratory, cardiovascular, endocrine, neurological, or hematological disease.

A total of 26 subjects with BD were recruited for this study. To ensure that only BD subjects who had been exposed to lithium for a period of time sufficient to assess response were included in the gene-expression analysis, an *a priori* decision was made to include only those subjects who completed at least one month of treatment. A total of 20 subjects were included; one subject was withdrawn due to previously undiagnosed hypothyroidism, and five subjects dropped out of treatment without completing one month of treatment. Demographic and clinical information for the 20 subjects included in the study is summarized in Table 1.

**Table 1:** Study Participants- summary of demographic and clinical information

**Gender**	**Lithium Responders**	**Lithium Non-Responders**
	**N (%)**	**N (%)**
Male	2 (20)	4 (40)
Female	8 (80)	6 (60)
Ethnicity		
Caucasian	7 (70)	9 (90)
Non-caucasian	3 (30)	1 (10)
Bipolar I disorder	7 (70)	10 (100)
	**Mean ± SD**	**Mean ± SD**
Age	34.5 ± 10.6	42.0 ± 6.6
HAM-D (at entry)	26.2 ± 15.9	29.1 ± 14.8
HAM-A (at entry)	14.4 ± 8.6	14.7 ± 7.0
MADRS (at entry)	17.9 ± 11.6	20.7 ± 11.5
YMRS (at entry)	6.3 ± 5.8	5.9 ± 4.3
Lithium Level (average)	0.63 ± 0.14	0.65 ± 0.28

HAM-D = Hamilton Depression Rating Scale, HAM-A = Hamilton Anxiety Rating Scale, MADRS = Montgomery-Asberg Depression Rating Scale, YMRS = Young Mania Rating Scale.

An additional group of 15 healthy control subjects (5 male, 10 female) was recruited through advertising. None of the control subjects met criteria for any DSM-IV-TR axis I diagnosis as determined by the Structured Clinical Interview for DSM-IV Axis I Disorders [24] or current or recent abuse of illicit substances.

### Treatments

All subjects received open label treatment with Lithium carbonate in addition to their previous psychiatric medications. Lithium was started at an initial dose of 300 mg p.o. BID. Doses were adjusted weekly based on lithium trough levels until a target level of 0.6 to 1.2 mEq/L was achieved or patients were unable to tolerate side effects. Of the 20 subjects included in the microarray analysis, there were 9 patients who were initially receiving no medication and 11 who were receiving one or more atypical antipsychotic medications (olanzapine, quietapine, risperidone, or ziprasidone). Two subjects were taking valproic acid in addition to an atypical antipsychotic and one subject each was taking carbamazepine, oxcarbazepine or topiramate in addition to an atypical antipsychotic. Mood ratings for BD subjects were performed using Hamilton Depression Rating Scale (HAM-D) [25,26], the Montgomery-Asberg Depression Rating Scale (MADRS) [27], Hamilton Anxiety Rating Scale [28], and the Young Mania Rating Scale (YMRS) [29].

### Sample Preparation and Microarray Analysis

Blood draws for RNA isolation were done prior to initiation of treatment with lithium and every two weeks during 8-weeks of open label treatment with lithium for subjects with BD (five blood draws total). Blood draws for RNA isolation were done at the same time as those used to assess lithium trough levels, approximately 12 hours after the evening dose of lithium, and before the morning dose was taken. Total RNA was isolated from 10 cc whole blood using the PAXgene Blood RNA Isolation kit (QIAGEN, Valencia, CA) per the manufacturer's instructions, and depleted of globin mRNA message using GLOBINclear hybridization capture technology (Ambion, Austin, TX). Globin-reduced total RNA underwent cDNA synthesis and overnight *in vitro transcription* utilizing the Illumina TotalPrep RNA Amplification Kit (Ambion). Biotinylated cRNA (1.5 μg) was hybridized onto an Illumina Sentrix Beadchip (Human-6v2) then scanned on a BeadArray Reader. Microarray hybridization and scanning were carried out at the NIH Neuroscience Microarray Center at Yale (http:/info.med.yale.edu/neuromicroarray). Per the policies of the NIH microarray consortium, the complete project annotation in MAGE-ML, image files, as well as raw data files will be available for download. At the time of publication, all data will be deposited into the NCBI-GEO repository, while retaining links to the microarray consortium relational data warehouse.

### Data Analysis

BD subjects were divided into lithium-responders and non-responders based on the *a priori* defined change from their initial HAM-D scores. Lithium-responders were defined as those having a >50% reduction in initial HAM-D at the time of the last assessment. BD subjects who did not meet these criteria were classified as "non-responders". Subjects who dropped out during weeks 4–8 were classified as lithium-responders or non-responders using an intent-to-treat analysis based on the last observation carried forward. Classification of BD subjects as lithium-responders vs. non-responders did not change if the MADRS was used instead of the HAM-D to classify subjects.

Statistical analysis of microarray data was carried out at the Keck Foundation Biotechnology Biostatistics Resource (http://keck.med.yale.edu/biostats). Illumina BeadStudio software was used to generate probe and gene expression profiles of each sample. Quantile normalization was carried out using the package incorporated in the Illumina BeadStudio software package [30]. Further statistical analysis was carried out on genes with a detection p-value <0.01 as determined using the Illumina BeadStudio software (i.e. a 99% probability that expression was above background) in 90% of the samples. A total of 17,240 genes on the array met these criteria. This is similar to the detection sensitivity seen in other studies of whole blood using the Illumina Sentrix Beadchip platform [31].

Baseline differences in gene-expression between lithium-responders and non-responders were assessed using t-tests as well as ANOVA analysis co-varied for age, sex, and co-administered medications. To identify genes whose expression changed differentially in lithium responders and non-responders after initiation of treatment, we performed a mixed model ANOVA of the complete microarray data set with group (responder or non-responder) as a between subjects factor, and time as a within subjects factor. Correction for multiple testing was done using estimated group-wise false discovery rates (FDR) [32,33].

Network analysis to identify the most significant pathways among genes identified by the ANOVA analysis (above) was carried out using GeneGO Metacore® software (GeneGO Inc., Encinitas, CA).

### qRT-PCR analysis

qRT-PCR was carried out using the TaqMan® "Universal PCR Master Mix" Protocol (Applied Biosystems) and Real-Time PCR probes listed on the NCBI Probe Database (http://www.ncbi.nlm.nih.gov/sites/entrez?db=probe). Relative quantitation of gene-expression was done by comparing the efficiency of amplification of each gene of interest using the ΔΔCt method, as described in User Bulletin#2 for the ABI Prism 7700 Sequence Detection System (Applied Biosystems, available online at http://keck.med.yale.edu/affymetrix/rtpcr/index.htm).

## Results

Lithium responders did not differ significantly from non-responders in their initial HAM-D, HAM-A, MADRS or YMRS scores, age, sex, ethnicity, or use of concomitant antipsychotic medication, however there was a non-significant trend for greater use of antipsychotic medications among the lithium non-responders. Average serum levels of lithium over the eight weeks of treatment did not differ significantly between responders and non-responders (responders: 0.63 ± 0.14; non-responders: 0.66 ± 0.28). Comparison of baseline (pre-treatment) expression profiles between lithium responders and non-responders using t-tests identified 606 genes, whose expression differed by ≥ 1.3 fold with a nominal p-value <0.05. However, when other factors such as age, sex, and use of co-administered medications were included as co-variates in an ANOVA model, none of these differences were significant after correction for multiple testing (FDR <0.05) (data not shown). Thus, it is unclear whether any of these pre-treatment differences are specifically associated with the subsequent response to lithium.

Next, to identify genes whose expression changed differentially in lithium responders and non-responders after initiation of treatment, we performed a mixed model ANOVA of the complete microarray data set with group (responder or non-responder) as a between subjects factor, and time (in weeks, after starting treatment with lithium) as a within subjects factor. There were 127 genes that showed a significant group x time interaction (i.e. difference in degree or direction of change between lithium-responders and non-responders) after FDR correction for multiple testing, and a fold-difference ≥ 1.3 between lithium responders and non-responders at least one time-point after treatment initiation. Interestingly, all of the significant differences between responders and non-responders occurred during the period from 4–6 weeks after initiation of treatment with lithium. This time period corresponds well with the typical 6–8-week delay in the acute antidepressant effect of lithium in the treatment of bipolar depression [34]. At week 4 there were 37 differentially expressed genes (22 up-regulated in responders vs. non-responders and 15 down-regulated) and at week 6 there were 90 differentially expressed genes (51 up-regulated and 39 down-regulated). The complete list of genes showing a significant group x time interaction is listed in Additional file 1 Table S1.

To better understand the functional implications of these differences, we conducted pathway analysis using GeneGO Metacore software for each of these clusters separately, and for the group of 127 genes as a whole. As seen in Table 2, 4 of the top 10 GeneGO Process Networks associated with the group as a whole are related to the regulation of apoptosis, although most of these pathways did not reach statistical significance. Moreover, when considered separately, each of the clusters of differentially expressed genes was related to one or more apoptotic pathways, although again, many of these pathways did not reach statistical significance. Notably, at 4 weeks after treatment initiation, pro-apoptotic mitochondrial genes appears to be down-regulated, while anti-apoptosis pathways regulated by external signals via PI3K/AKT appeared to be up-regulated in lithium-responders vs. non-responders. Based on these findings, as well as previous work implicating the Bcl2 family of proteins in the mechanism of action of lithium [17,18], we decided to examine the pattern of expression of various Bcl2 gene family members over time in lithium-responders and non-responders more closely.

**Table 2:** Biological pathways identified by GeneGO metacore pathway analysis as differing over time between BD subjects who responded or failed to respond to treatment with lithium

**GeneGO Process Networks for genes showing significant group x time interaction**		
**Overall**		**p-Value**
1	Apoptosis_Anti-Apoptosis mediated by external signals	0.013
2	Apoptosis_Apoptotic mitochondria	0.055
3	Cytoskeleton_Actin filaments	0.055
4	Apoptosis_Endoplasmic reticulum stress pathway	0.068
5	Cell cycle_Core	0.110
6	Protein folding_Folding in normal condition	0.117
7	Cell cycle_G1-S Interleukin regulation	0.123
8	Development_Hemopoiesis, Erythropoietin pathway	0.143
9	Immune response_BCR pathway	0.147
10	Apoptosis_Apoptosis stimulation by external signals	0.159
**Week 4: down-regulated genes in Lithium responders vs. non-responders**		
1	Apoptosis_Apoptotic mitochondria	0.053
2	Apoptosis_Endoplasmic reticulum stress pathway	0.060
3	Signal transduction_Nitric oxide signaling	0.061
4	Apoptosis_Anti-Apoptosis mediated by external signals by Estrogen	0.068
5	Protein folding_Folding in normal condition	0.081
6	Proteolysis_Proteolysis in cell cycle and apoptosis	0.085
7	Development_Hemopoiesis, Erythropoietin pathway	0.092
8	Apoptosis_Apoptosis stimulation by external signals	0.098
9	Proliferation_Negative regulation of cell proliferation	0.123
10	Cell adhesion_Amyloid proteins	0.127
**Week 4: up-regulated genes in Lithium responders vs. non-responders**		
1	Cell adhesion_Glycoconjugates	0.010
2	Apoptosis_Anti-Apoptosis mediated by external signals via PI3K/AKT	0.017
3	Autophagy_Autophagy	0.053
4	Signal transduction_Androgen receptor signaling cross-talk	0.069
5	Signal transduction_ERBB-family signaling	0.072
6	Cytoskeleton_Macropinocytosis and its regulation	0.081
7	Inflammation_IL-13 signaling pathway	0.087
8	Signal transduction_Leptin signaling	0.098
9	Signal Transduction_Cholecystokinin signaling	0.100
10	Inflammation_IL-4 signaling	0.108
**Week 6: down-regulated genes in Lithium responders vs. non-responders**		
1	Cell cycle_Core	0.027
2	Development_Hemopoiesis, Erythropoietin pathway	0.036
3	Response to hypoxia and oxidative stress	0.053
4	Transport_Synaptic vesicle exocytosis	0.059
5	Cell cycle_G0-G1	0.149
6	Neurophysiological process_Long-term potentiation	0.170
7	Blood coagulation	0.193
8	Apoptosis_Anti-Apoptosis mediated by external signals by Estrogen	0.202
9	Inflammation_IL-6 signaling	0.238
10	Cell cycle_G1-S Interleukin regulation	0.245
**Week 6: up-regulated genes in Lithium responders vs. non-responders**		
1	Development_Skeletal muscle development	0.024
2	Cell adhesion_Attractive and repulsive receptors	0.034
3	Cytoskeleton_Actin filaments	0.034
4	Development_Neurogenesis:Axonal guidance	0.056
5	Protein folding_ER and cytoplasm	0.074
6	Protein folding_Response to unfolded proteins	0.111
7	Apoptosis_Apoptotic mitochondria	0.123
8	Apoptosis_Endoplasmic reticulum stress pathway	0.138
9	Apoptosis_Anti-Apoptosis mediated by external signals by Estrogen	0.156
10	Protein folding_Folding in normal condition	0.184

Pathway analysis was conducted for the entire group of 127 genes showing a significant group x time interaction, as well as for up-regulated and down-regulated genes separately at each of the time-points where significant differences were seen (4 weeks and 6 weeks after treatment initiation).

**Figure 1:**
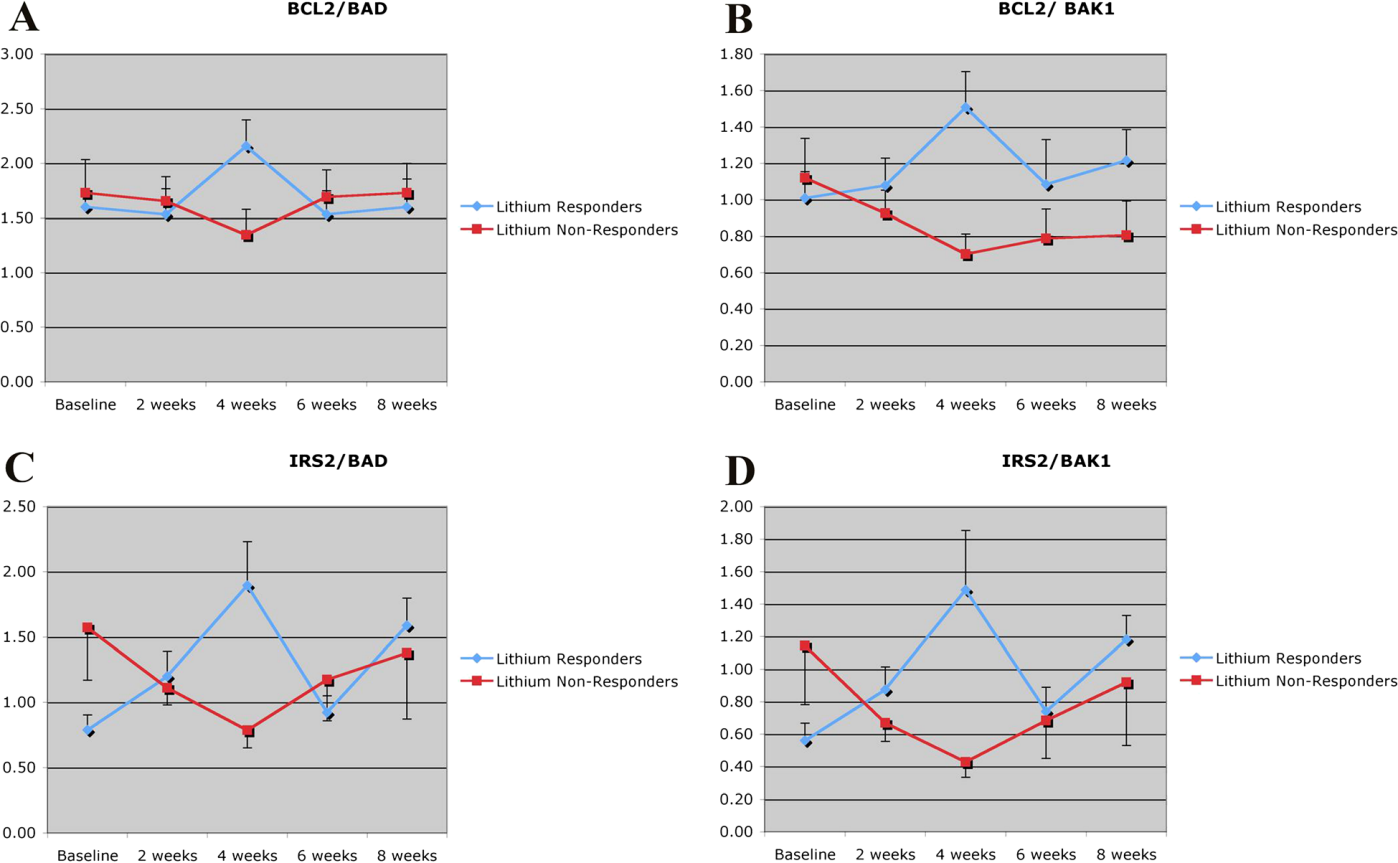
**Ratios of BCL2/ BAD (panel A), BCL2/BAK1 (panel B), IRS2/BAD (panel C) and IRS2/BAK1 (panel D) in lithium responders and non-responders over the 8 weeks of the study**. In each case, the ratio of anti- to pro-apoptotic genes increased in lithium responders over the first month of treatment and then returned to baseline, while the opposite pattern was observed in lithium non-responders.

Table 3 shows the expression level of various pro- and anti-apoptotic Bcl2 gene family member expression in lithium-responders and non-responders (normalized to healthy controls) as well as the ratio between responders and non-responders over the course of eight weeks following initiation of treatment with lithium. Strikingly, following initiation of treatment with lithium, all of the anti-apoptotic genes examined (BCL2, BCL2L1-tx var. 1, IRS2, and MCL1- tx. var. 1), showed an increase in relative expression in lithium responders compared to non-responders during the first month of treatment. For BCL2L1, this increase peaked at 2 weeks after treatment initiation, while for the other genes the highest relative expression occurred at 4 weeks. Among the pro-apoptotic genes, BAD, BAK, BAX, and BMF showed a decrease in the relative expression in lithium responders, while BCL2L13, BCL2L1-tx. var. 2, BID, BNIP3, and MCL1, tx. var. 2, showed no change or inconsistent change over this time period. Thus, overall there appeared to be an increase in the relative expression of anti-apoptotic genes and a decrease in the expression of pro-apoptotic genes in lithium responders during the first month of treatment, while the opposite pattern was seen in lithium non-responders. This pattern was even more marked when the ratios of specific anti- and pro-apoptotic genes were compared in lithium responders and non-responders over time. Figure 1 shows the ratios of BCL2/ BAD (panel **A**), BCL2/BAK1 (panel **B**), IRS2/BAD (panel **C)** and IRS2/BAK1 (panel **D**) in lithium responders and non-responders over the 8 weeks of the study. In each case, the ratio of anti- to pro-apoptotic genes increased in lithium responders over the first month of treatment and then returned to baseline, while the opposite pattern was observed in lithium non-responders. When the ratios of alternatively spliced versions of the same gene with either anti- or pro- apoptotic functions (e.g. BCL2L1 tx. variants 1 and 2, or MCL1 tx. variants 1 and 2) were compared, there was no significant group x time interaction, indicating that alternative splicing was not responsible for these effects.

**Table 3:** Expression of anti- and pro-apoptotic Bcl2-related genes in lithium responders and non-responders (normalized to expression levels in untreated healthy controls subjects) at each of the time points tested (baseline, 2,4,6, and 8 weeks after treatment initiation)

**Normalized Expression Levels**	**Time (in weeks) since starting lithium**				
**Anti-Apoptotic Genes**	**Baseline**	**2 weeks**	**4 weeks**	**6 weeks**	**8 weeks**
**BCL2**					
Lithium Responders	0.96	0.89	1.08	0.86	0.83
Non-Responders	0.94	0.90	0.78	0.88	0.93
Ratio	1.0	1.0	*1.4	1.0	0.9
**BCL2L1 (tx var. 1) (aka BCL-xL)**					
Lithium Responders	2.30	4.69	2.38	2.49	5.84
Non-Responders	3.04	3.49	2.08	3.13	1.85
Ratio	0.8	1.3	1.1	0.8	3.2
**IRS2**					
Lithium Responders	0.78	1.12	1.51	0.84	1.35
Non-Responders	1.22	0.99	0.68	1.00	1.13
Ratio	0.6	1.1	***2.2	0.8	1.2
**MCL1 (tx. Var. 1)**					
Lithium Responders	0.56	0.81	0.91	0.74	0.98
Non-Responders	1.18	1.00	0.79	1.02	1.05
Ratio	**0.5	0.8	1.1	0.7	0.9
**Pro-Apoptotic Genes**					
**BAD**					
Lithium Responders	1.54	1.49	1.29	1.44	1.32
Non-Responders	1.39	1.39	1.48	1.33	1.41
Ratio	1.1	1.1	0.9	1.1	0.9
**BAK1**					
Lithium Responders	1.47	0.92	0.80	0.89	0.76
Non-Responders	1.29	1.08	1.23	1.24	1.29
Ratio	1.1	0.9	***0.6	**0.7	0.6
**BAX**					
Lithium Responders	1.69	1.73	1.55	1.58	1.18
Non-Responders	1.14	1.39	1.25	1.42	1.53
Ratio	**1.5	1.2	1.2	1.1	0.8
**BCL2L13 (aka BCL-Rambo)**					
Lithium Responders	0.86	0.98	0.96	0.82	0.96
Non-Responders	0.96	1.03	0.86	0.97	0.85
Ratio	0.9	0.9	1.1	0.8	1.1
**BCL2L1 (tx var. 2)(aka BCL-xL)**					
Lithium Responders	1.58	2.34	1.54	1.42	1.54
Non-Responders	1.58	1.98	1.59	1.96	1.36
Ratio	1.0	1.2	1.0	0.7	1.1
**BID**					
Lithium Responders	1.03	1.06	1.09	0.98	1.01
Non-Responders	1.04	0.99	1.02	1.12	0.97
Ratio	1.0	1.1	1.1	0.9	1.0
**BMF**					
Lithium Responders	1.09	1.05	1.06	1.06	0.96
Non-Responders	0.91	0.97	1.10	0.82	1.06
Ratio	1.2	1.1	1.0	*1.3	0.9
**BNIP3**					
Lithium Responders	1.02	0.74	0.89	0.78	0.78
Non-Responders	0.81	0.84	0.77	0.82	0.91
Ratio	**1.3	0.9	1.2	0.9	0.9
**MCL1 (tx. var. 2)**					
Lithium Responders	1.08	1.21	1.14	1.10	1.34
Non-Responders	1.11	1.24	1.10	1.19	1.02
Ratio	1.0	1.0	1.0	0.9	*1.3

Following initiation of treatment with lithium, all of the anti-apoptotic genes examined (BCL2, BCL2L1-tx var. 1, IRS2, and MCL1- tx. var. 1), showed an increase in the relative expression in lithium responders compared to non-responders during the first month of treatment. Among the pro-apoptotic genes, BAD, BAK, BAX, and BMF showed a decrease in the relative expression in lithium responders, while BCL2L13, BCL2L1-tx. var. 2, BID, BNIP3, and MCL1, tx. var. 2, showed no change or inconsistent change over this time period.

Significant differences are marked with asterisks (* p <0.10, ** p <0.05, and *** P < .01).

We also performed qRT-PCR analysis of the 4 genes shown in Figure 1: BCL2, IRS2, BAK1 and BAD, and compared the ratio of anti-apoptotic to pro-apoptotic gene expression in lithium responders and non-responders 4 weeks after treatment initiation. In general, the results of the qRT-PCR analysis were similar to those obtained by microarray hybridization, with lithium-responders showing greater relative expression of anti-apoptotic genes than non-responders (BCL2/BAK1: 1.37 fold higher in responders vs. non-responders, BLC2/BAD: 1.69 fold higher in responders vs. non-responders, IRS2/BAK1: 1.04 fold higher in responders vs. non-responders, IRS2/BAD: 1.29 fold higher in responders vs. non-responders). However, due to the greater variability in the qRT-PCR results, none of these differences was statistically significant.

## Discussion

In this study, we compared changes in gene-expression in peripheral blood among a group of depressed subjects with BD over a period of eight weeks following the initiation of treatment with lithium. We identified 127 genes whose expression changed differentially in lithium responders and non-responders. Pathway analysis of the differentially expressed genes using GeneGO Metacore software showed that regulation of apoptosis was the most significantly affected pathway among these genes. Strikingly, among lithium responders, several anti-apoptotic genes including BCL2 and insulin receptor substrate 2 (IRS2) were up-regulated, while pro-apoptotic genes, including BCL2-antagonist/killer 1 (BAK1) and BCL2-associated agonist of cell death (BAD), were down-regulated. In contrast, in lithium non-responders, BCL2 and IRS2 were down-regulated, while BAK1 and BAD up-regulated at the one-month time-point.

BCL2 gene family members are key regulators of apoptotic cell death and include both pro- and anti-apoptotic genes [35,36]. Collectively, the expression levels of the various BCL2 family members define thresholds for apoptosis in a given cell. The two pro-apoptotic proteins, BAX and BAK1 promote apoptosis by binding to the mitochondrial voltage-dependent anion channel (VDAC), and accelerating its opening, leading to a loss in membrane potential, the release of cytochrome c, and subsequent activation of the intrinsic caspase pathway. Anti-apoptotic members of the BCL2 family, including BCL2 itself, the related protein BCL-xL (encoded by the BLCL1 gene) and MCL-1, prevent apoptosis by binding to BAX and BAK1, preventing their interaction with VDAC. Other pro-apoptotic BCL2 family members, termed the ‘BH3-only’ proteins, including BAD, BID, and BMF, are thought to indirectly promote apoptosis by binding to anti-apoptotic family members and preventing their interaction with BAX and BAK1 [35,36]. IRS2 is a cytoplasmic signaling molecule that mediates effects of insulin, insulin-like growth factor 1, and other cytokines by acting as a molecular adaptor between diverse receptor tyrosine kinases and downstream effectors. In addition, IRS2 has been shown to bind to the Bcl2 protein and block phosphorylation of Bcl2 induced by insulin and suppress apoptotic cell death [37]. Thus, our finding that BCL2 and IRS2 are both increased in lithium responders at the one month time point, while BAK1 and BAD were down-regulated, suggests that in lithium responders there was a shift in the balance of expression among pro- and anti- apoptotic members of the BCL2 family favoring the anti-apoptotic genes. Conversely, in lithium non-responders there was a decrease in BCL2 and IRS2 and an increase BAK1 and BAD, suggesting that there was a shift in the opposite direction, favoring the pro-apoptotic members of the BCL2 family. Intriguingly, changes in the ratio of anti- to pro-apoptotic gene expression among both lithium responders and non-responders appeared to return to baseline by the 8-week time-point, although differences in clinical status were more marked at this point than at 4 weeks. This suggests that transient changes in gene-expression can have enduring effects on the state of the organism, even when those differences can no longer be directly observed. Additional studies will be required to determine if there are changes in the level or function of the proteins encoded by these genes that are longer lasting and perhaps more directly related to the clinical status of the patients.

While changes in the expression of BCL2 family genes in peripheral blood are unlikely to be directly related to the changes in mood symptoms, systemic differences in the way subjects with different genetic and epigenetic backgrounds respond at the biochemical level to treatment with lithium may underlie some of the heterogeneity in clinical response to lithium.

Studies of post-mortem tissue from human subjects has shown that activity of mitochondrial complex I is decreased, and oxidative damage is increased in the prefrontal cortex of patients with BD [38]. Lithium has been shown to increase the activity of mitochondrial ETC. complexes in extracts from human post-mortem brain tissue at therapeutically relevant concentrations [39], while rats subjected to an experimental model of depression showed impaired mitochondrial function [40]. Conversely, transgenic mice expressing the anti-apoptotic protein Bax Inhibitor 1, showed protection in the learned helplessness model of depression [41]. These results have been interpreted in the context of a neurotrophic hypothesis of mood disorders [42,43], indicating that increased expression of BCL2 and related genes is necessary for the therapeutic effects of lithium and other mood stabilizers. Our results indicate that in a substantial group of patients, this effect does not occur, and in fact the opposite effect was seen in patients who did not respond to lithium. Better understanding of the mechanisms underlying this difference may lead to improved methods for personalizing treatment for bipolar disorder in the future.

Limitations of this study include a relatively small sample-size, admixture of subjects with BD I and BD II, and the fact that BCL2 family members were assayed at the level of gene-expression rather than protein or functional assays. A further caveat is that while the average Li level in both groups was similar, and was within our target range of 0.6 to 1.2 mEq/L, there were several subjects in both groups whose levels were outside of that range, which may have affected both their response to treatment and the patterns of gene-induction observed in these subjects. In addition, these studies address only the acute anti-depressant properties of lithium, and do not address changes in gene-expression that may be related to lithium’s anti-manic, prophylactic or anti-suicide properties, each of which may be associated with a unique molecular profile. Due to the small sample size and limited information that was collected regarding prior episodes, we are unable to address the relationship, if any, between these molecular changes and the proposed specificity of lithium for “classic bipolar disorder” as opposed to the broader spectrum of bipolar illnesses [8]. Future studies will be needed to confirm these findings in a larger cohort of patients and to determine the relevancy of these changes to other aspects of lithium’s clinical effects in patients with BD.

## Conclusions

In this study, we compared changes in gene-expression in peripheral blood among a group of depressed subjects with BD over a period of eight weeks following the initiation of treatment with lithium. We found that the ratio of anti- to pro-apoptotic gene expression increased in lithium responders over the first month of treatment and then returned to baseline, while the opposite pattern was observed in lithium non-responders. These results suggest that individual differences in the response to treatment with lithium occur at the level of gene-induction, and are clinically relevant. If validated in larger studies, such changes could be useful clinically as surrogate outcome markers allowing treatment decisions (including whether to continue or discontinue treatment with lithium) to be made earlier, and thus facilitate recovery in patients with BD.

## Competing interests

The authors declare that they have no competing interests.

## Authors’ contributions

LL participated in the design of the study, carried out RT-PCR analyses of candidate mRNAs and wrote the initial draft of the manuscript. JL performed RNA isolation from blood samples and maintained the laboratory database of samples. AL carried out statistical analyses of microarray and clinical data. SU carried out quality control on RNA samples and performed microarray hybridizations. KM carried out subject recruitment and assessment. AM carried out subject recruitment and assessment. BL carried out subject recruitment and assessment. SM participated in the design of the study and supervised microarray hybridizations. HZ participated in the design of the study and supervised the statistical analysis. RS participated in the design of the study, supervised recruitment and assessment of healthy controls. ZB participated in the design of the study, supervised subject recruitment and assessment, performed diagnostic interviews, and helped to draft the manuscript. RB conceived of the study, participated in its design and coordination, carried out diagnostic interviews and subjects assessments, and wrote the final draft of the mancuscript. All authors read and approved the final manuscript.

## Supplementary Material

Additional file 1 **Table S1.** Fold difference and p-value for each of 127 genes, grouped by cluster, that showed a significant group x time interaction (i.e. difference in degree or direction of change between lithium-responders and non-responders) after FDR correction for multiple testing , and a fold-difference ≥ 1.3 between lithium responders and non-responders at least one time-point after treatment initiation. Click here for file
