# Supplementary material for: Increased ratio of anti-apoptotic to pro-apoptotic Bcl2 gene-family members in lithium-responders one month after treatment initiation
Source: Biol Mood Anxiety Disord. 2012 Sep 12;2:15. doi: 10.1186/2045-5380-2-15 (PMC3448519; doi:10.1186/2045-5380-2-15)
Supplement: Additional file 1 — Table S1. Fold difference and p-value for each of 127 genes, grouped by cluster, that showed a significant group x time interaction (i.e. difference in degree or direction of change between lithium-responders and non-responders) after FDR correction for multiple testing , and a fold-difference ≥ 1.3 between lithium responders and non-responders at least one time-point after treatment initiation. [file 2045-5380-2-15-S1.docx]

| Cluster 1 (Increased in Responder vs. Non-responder at 4 weeks) | | | | |
| --- | --- | --- | --- | --- |
| Genbank | Gene Symbol | Description | p-value (Responder vs. Non- responder at 4 weeks) | Fold-change (Responder vs. Non- responder at 4 weeks) |
| NM_003749.2 | IRS2 | Homo sapiens insulin receptor substrate 2 (IRS2), mRNA. | 0.004 | **2.14** |
| NM_172345.1 | SPAG9 | Homo sapiens sperm associated antigen 9 (SPAG9), transcript variant 2, mRNA. | 0.004 | **1.89** |
| NM_003980.3 | MAP7 | Homo sapiens microtubule-associated protein 7 (MAP7), mRNA. | 0.003 | **1.85** |
| NM_013314.2 | BLNK | Homo sapiens B-cell linker (BLNK), mRNA. | 0.003 | **1.71** |
| BX111822 |  | BX111822 Soares fetal liver spleen 1NFLS Homo sapiens cDNA clone IMAGp998M11419, mRNA sequence | 0.001 | **1.71** |
| NM_001005409.1 | SF3A1 | Homo sapiens splicing factor 3a, subunit 1, 120kDa (SF3A1), transcript variant 2, mRNA. | 0.001 | **1.71** |
| NM_031305.2 | ARHGAP24 | Homo sapiens Rho GTPase activating protein 24 (ARHGAP24), transcript variant 2, mRNA. | 0.003 | **1.68** |
| XM_937058.1 | ZNF654 | PREDICTED: Homo sapiens zinc finger protein 654 (ZNF654), mRNA. | 0.001 | **1.67** |
| NM_001656.3 | TRIM23 | Homo sapiens tripartite motif-containing 23 (TRIM23), transcript variant alpha, mRNA. | 0.003 | **1.66** |
| NM_006506.2 | RASA2 | Homo sapiens RAS p21 protein activator 2 (RASA2), mRNA. | 0.001 | **1.65** |
| NM_022913.1 | GPBP1 | Homo sapiens GC-rich promoter binding protein 1 (GPBP1), mRNA. | 0.003 | **1.61** |
| NM_018017.2 | C10orf118 | Homo sapiens chromosome 10 open reading frame 118 (C10orf118), mRNA. | 0.002 | **1.61** |
| NM_020169.2 | LXN | Homo sapiens latexin (LXN), mRNA. | 0.002 | **1.50** |
| NM_005327.2 | HADH | Homo sapiens hydroxyacyl-Coenzyme A dehydrogenase (HADH), nuclear gene encoding mitochondrial protein, mRNA. | 0.003 | **1.41** |
| XM_371542.4 | RW1 | PREDICTED: Homo sapiens RW1 protein, transcript variant 1 (RW1), mRNA. | 0.003 | **1.41** |
| NM_014824.1 | FCHSD2 | Homo sapiens FCH and double SH3 domains 2 (FCHSD2), mRNA. | 0.001 | **1.40** |
| NM_152334.2 | TARSL2 | Homo sapiens threonyl-tRNA synthetase-like 2 (TARSL2), mRNA. | 0.004 | **1.38** |
| XM_936978.1 | ZNF136 | PREDICTED: Homo sapiens zinc finger protein 136 (clone pHZ-20) (ZNF136), mRNA. | 0.002 | **1.37** |
| XM_937221.1 | LOC648176 | PREDICTED: Homo sapiens similar to RAS and EF hand domain containing (LOC648176), mRNA. | 0.001 | **1.36** |
| NM_014613.1 | UBXD8 | Homo sapiens UBX domain containing 8 (UBXD8), mRNA. | 0.002 | **1.35** |
| NM_018469.3 | TEX2 | Homo sapiens testis expressed sequence 2 (TEX2), mRNA. | 0.003 | **1.34** |
| NM_032431.2 | SYVN1 | Homo sapiens synovial apoptosis inhibitor 1, synoviolin (SYVN1), transcript variant 1, mRNA. | 0.000 | **1.32** |

| Cluster 2 (Decreased in Responder vs. Non-responder at 4 weeks) | | | | |
| --- | --- | --- | --- | --- |
| Genbank | Gene Symbol | Description | p-value (Responder vs. Non- responder at 4 weeks) | Fold-change (Responder vs. Non- responder at 4 weeks) |
| NM_024660.1 | U2AF1L4 | Homo sapiens U2(RNU2) small nuclear RNA auxiliary factor 1-like 4 (U2AF1L4), mRNA. | 0.003 | **-1.31** |
| NM_004809.3 | STOML1 | Homo sapiens stomatin (EPB72)-like 1 (STOML1), mRNA. | 0.001 | **-1.35** |
| NM_172341.1 | PSENEN | Homo sapiens presenilin enhancer 2 homolog (C. elegans) (PSENEN), mRNA. | 0.000 | **-1.38** |
| NM_032630.2 | CINP | Homo sapiens cyclin-dependent kinase 2-interacting protein (CINP), mRNA. | 0.000 | **-1.39** |
| NM_181301.1 | CMTM1 | Homo sapiens CKLF-like MARVEL transmembrane domain containing 1 (CMTM1), transcript variant 23, mRNA. | 0.001 | **-1.39** |
| NM_057174.1 | PEX16 | Homo sapiens peroxisomal biogenesis factor 16 (PEX16), transcript variant 2, mRNA. | 0.000 | **-1.44** |
| NM_014260.2 | PFDN6 | Homo sapiens prefoldin subunit 6 (PFDN6), mRNA. | 0.002 | **-1.49** |
| NM_004913.2 | C16orf7 | Homo sapiens chromosome 16 open reading frame 7 (C16orf7), mRNA. | 0.001 | **-1.50** |
| NM_014297.3 | ETHE1 | Homo sapiens ethylmalonic encephalopathy 1 (ETHE1), mRNA. | 0.002 | **-1.52** |
| NM_001188.3 | BAK1 | Homo sapiens BCL2-antagonist/killer 1 (BAK1), mRNA. | 0.001 | **-1.55** |
| NM_031300.2 | MXD3 | Homo sapiens MAX dimerization protein 3 (MXD3), mRNA. | 0.000 | **-1.57** |
| NM_001012715.1 | C9orf106 | Homo sapiens chromosome 9 open reading frame 106 (C9orf106), mRNA. | 0.004 | **-1.59** |
| NM_020415.2 | RETN | Homo sapiens resistin (RETN), mRNA. | 0.002 | **-1.75** |
| BF508745 |  | UI-H-BI4-aoq-c-11-0-UI.s1 NCI_CGAP_Sub8 Homo sapiens cDNA clone IMAGE:3085820 3, mRNA sequence | 0.000 | **-2.13** |
| NM_005332.2 | HBZ | Homo sapiens hemoglobin, zeta (HBZ), mRNA. | 0.000 | **-3.14** |

| Cluster 3 (Increased in Responder vs. Non-responder at 6 weeks) FDR <0.05 | | | | |
| --- | --- | --- | --- | --- |
| Genbank | Gene Symbol | Description | p-value (Responder vs. Non- responder at 6 weeks) | Fold-change (Responder vs. Non- responder at 6 weeks) |
| BE819416 |  | RC0-BN0329-210600-031-f07 BN0329 Homo sapiens cDNA, mRNA sequence | 0.000 | **2.23** |
| NM_005560.3 | LAMA5 | Homo sapiens laminin, alpha 5 (LAMA5), mRNA. | 0.000 | **2.12** |
| NM_032592.1 | PHACS | Homo sapiens 1-aminocyclopropane-1-carboxylate synthase (PHACS), mRNA. | 0.000 | **1.94** |
| NM_003289.3 | TPM2 | Homo sapiens tropomyosin 2 (beta) (TPM2), transcript variant 1, mRNA. | 0.000 | **1.93** |
| NM_198075.1 | LRRC56 | Homo sapiens leucine rich repeat containing 56 (LRRC56), mRNA. | 0.000 | **1.89** |
| NM_001783.3 | CD79A | Homo sapiens CD79a molecule, immunoglobulin-associated alpha (CD79A), transcript variant 1, mRNA. | 0.003 | **1.84** |
| NM_021966.1 | TCL1A | Homo sapiens T-cell leukemia/lymphoma 1A (TCL1A), mRNA. | 0.003 | **1.80** |
| NM_006647.1 | NOXA1 | Homo sapiens NADPH oxidase activator 1 (NOXA1), mRNA. | 0.000 | **1.71** |
| NM_175907.3 | ZADH2 | Homo sapiens zinc binding alcohol dehydrogenase, domain containing 2 (ZADH2), mRNA. | 0.001 | **1.68** |
| XR_015544.1 | LOC202181 | PREDICTED: Homo sapiens hypothetical protein LOC202181 (LOC202181), misc RNA. | 0.000 | **1.63** |
| NM_000156.4 | GAMT | Homo sapiens guanidinoacetate N-methyltransferase (GAMT), transcript variant 1, mRNA. | 0.001 | **1.63** |
| NM_001008701.1 | LPHN1 | Homo sapiens latrophilin 1 (LPHN1), transcript variant 1, mRNA. | 0.000 | **1.60** |
| NM_138352.1 | SAMD1 | Homo sapiens sterile alpha motif domain containing 1 (SAMD1), mRNA. | 0.004 | **1.59** |
| NM_006885.3 | ATBF1 | Homo sapiens AT-binding transcription factor 1 (ATBF1), mRNA. | 0.000 | **1.58** |
| NM_014974.1 | DIP2C | Homo sapiens DIP2 disco-interacting protein 2 homolog C (Drosophila) (DIP2C), mRNA. | 0.002 | **1.58** |
| NM_001235.2 | SERPINH1 | Homo sapiens serpin peptidase inhibitor, clade H (heat shock protein 47), member 1, (collagen binding protein 1) (SERPINH1), mRNA. | 0.000 | **1.58** |
| NM_022370.2 | ROBO3 | Homo sapiens roundabout, axon guidance receptor, homolog 3 (Drosophila) (ROBO3), mRNA. | 0.000 | **1.57** |
| NM_144666.1 | DNHD1 | Homo sapiens dynein heavy chain domain 1 (DNHD1), mRNA. | 0.001 | **1.56** |
| XM_001127981.1 | LOC728014 | PREDICTED: Homo sapiens similar to huntingtin interacting protein 1 related (LOC728014), mRNA. | 0.001 | **1.56** |
| NM_173544.2 | FAM129C | Homo sapiens family with sequence similarity 129, member C (FAM129C), mRNA. | 0.007 | **1.56** |
| NM_014475.3 | DHDH | Homo sapiens dihydrodiol dehydrogenase (dimeric) (DHDH), mRNA. | 0.000 | **1.54** |
| NM_032242.2 | PLXNA1 | Homo sapiens plexin A1 (PLXNA1), mRNA. | 0.000 | **1.54** |
| XM_938582.1 | TMEM16J | PREDICTED: Homo sapiens transmembrane protein 16J (TMEM16J), mRNA. | 0.010 | **1.53** |
| XR_017982.1 | FLJ90757 | PREDICTED: Homo sapiens hypothetical protein LOC440465 (FLJ90757), misc RNA. | 0.001 | **1.52** |
| NM_016297.2 | PCYOX1 | Homo sapiens prenylcysteine oxidase 1 (PCYOX1), mRNA. | 0.001 | **1.52** |
| BX099724 |  | BX099724 Soares_fetal_liver_spleen_1NFLS_S1 Homo sapiens cDNA clone IMAGp998F201004, mRNA sequence | 0.004 | **1.51** |
| AW964566 |  | EST376639 MAGE resequences, MAGH Homo sapiens cDNA, mRNA sequence | 0.007 | **1.51** |
| NM_017773.2 | LAX1 | Homo sapiens lymphocyte transmembrane adaptor 1 (LAX1), mRNA. | 0.007 | **1.49** |
| NM_052847.1 | GNG7 | Homo sapiens guanine nucleotide binding protein (G protein), gamma 7 (GNG7), mRNA. | 0.001 | **1.49** |
| NM_018467.2 | MDS032 | Homo sapiens uncharacterized hematopoietic stem/progenitor cells protein MDS032 (MDS032), mRNA. | 0.004 | **1.49** |
| NM_017891.2 | C1orf159 | Homo sapiens chromosome 1 open reading frame 159 (C1orf159), mRNA. | 0.003 | **1.48** |
| NM_002120.3 | HLA-DOB | Homo sapiens major histocompatibility complex, class II, DO beta (HLA-DOB), mRNA. | 0.007 | **1.47** |
| NM_021646.1 | ZNF500 | Homo sapiens zinc finger protein 500 (ZNF500), mRNA. | 0.005 | **1.46** |
| XR_017956.1 | C6orf59 | PREDICTED: Homo sapiens chromosome 6 open reading frame 59 (C6orf59), misc RNA. | 0.010 | **1.44** |
| NM_015147.1 | CEP68 | Homo sapiens centrosomal protein 68kDa (CEP68), mRNA. | 0.010 | **1.43** |
| NM_001217.3 | CA11 | Homo sapiens carbonic anhydrase XI (CA11), mRNA. | 0.005 | **1.40** |
| NM_145738.1 | SYNGR1 | Homo sapiens synaptogyrin 1 (SYNGR1), transcript variant 1c, mRNA. | 0.006 | **1.40** |
| NM_144692.1 | LOC148137 | Homo sapiens hypothetical protein BC017947 (LOC148137), mRNA. | 0.002 | **1.39** |
| NM_006458.2 | TRIM3 | Homo sapiens tripartite motif-containing 3 (TRIM3), transcript variant 1, mRNA. | 0.003 | **1.39** |
| BQ720190 |  | AGENCOURT_8294407 Lupski_sympathetic_trunk Homo sapiens cDNA clone IMAGE:6194305 5, mRNA sequence | 0.002 | **1.38** |
| NM_004263.2 | SEMA4F | Homo sapiens sema domain, immunoglobulin domain (Ig), transmembrane domain (TM) and short cytoplasmic domain, (semaphorin) 4F (SEMA4F), mRNA. | 0.010 | **1.37** |
| NM_001089.2 | ABCA3 | Homo sapiens ATP-binding cassette, sub-family A (ABC1), member 3 (ABCA3), mRNA. | 0.006 | **1.37** |
| NM_022773.1 | FLJ12681 | Homo sapiens hypothetical protein FLJ12681 (FLJ12681), mRNA. | 0.008 | **1.36** |
| NM_015433.2 | FAM119B | Homo sapiens family with sequence similarity 119, member B (FAM119B), transcript variant 1, mRNA. | 0.010 | **1.35** |
| NM_032687.2 | CYHR1 | Homo sapiens cysteine/histidine-rich 1 (CYHR1), mRNA. | 0.003 | **1.35** |
| NM_022465.3 | IKZF4 | Homo sapiens IKAROS family zinc finger 4 (Eos) (IKZF4), mRNA. | 0.007 | **1.35** |
| NM_152742.1 | GPC2 | Homo sapiens glypican 2 (GPC2), mRNA. | 0.010 | **1.33** |
| BX113978 |  | BX113978 Soares_pregnant_uterus_NbHPU Homo sapiens cDNA clone IMAGp998N131206, mRNA sequence | 0.010 | **1.33** |
| NM_033503.3 | BMF | Homo sapiens Bcl2 modifying factor (BMF), transcript variant 2, mRNA. | 0.002 | **1.33** |
| NM_025082.3 | CENPT | Homo sapiens centromere protein T (CENPT), mRNA. | 0.009 | **1.32** |
| NM_003492.1 | CXorf12 | Homo sapiens chromosome X open reading frame 12 (CXorf12), mRNA. | 0.002 | **1.30** |

| Cluster 4 (Decreased in Responder vs. Non-responder at 6 weeks) | | | | |
| --- | --- | --- | --- | --- |
| Genbank | Gene Symbol | Description | p-value (Responder vs. Non- responder at 6 weeks) | Fold-change (Responder vs. Non- responder at 6 weeks) |
| NM_020548.5 | DBI | Homo sapiens diazepam binding inhibitor (GABA receptor modulator, acyl-Coenzyme A binding protein) (DBI), transcript variant 1, mRNA. | 0.005 | **-1.35** |
| NM_017662.3 | TRPM6 | Homo sapiens transient receptor potential cation channel, subfamily M, member 6 (TRPM6), mRNA. | 0.009 | **-1.35** |
| NM_005694.1 | COX17 | Homo sapiens COX17 cytochrome c oxidase assembly homolog (S. cerevisiae) (COX17), nuclear gene encoding mitochondrial protein, mRNA. | 0.007 | **-1.37** |
| NM_020382.3 | SETD8 | Homo sapiens SET domain containing (lysine methyltransferase) 8 (SETD8), mRNA. | 0.000 | **-1.40** |
| NM_144723.1 | ZMAT2 | Homo sapiens zinc finger, matrin type 2 (ZMAT2), mRNA. | 0.001 | **-1.42** |
| NM_016184.2 | CLEC4A | Homo sapiens C-type lectin domain family 4, member A (CLEC4A), transcript variant 1, mRNA. | 0.008 | **-1.46** |
| NM_006294.2 | UQCRB | Homo sapiens ubiquinol-cytochrome c reductase binding protein (UQCRB), mRNA. | 0.001 | **-1.47** |
| NM_079421.2 | CDKN2D | Homo sapiens cyclin-dependent kinase inhibitor 2D (p19, inhibits CDK4) (CDKN2D), transcript variant 2, mRNA. | 0.001 | **-1.48** |
| NM_003329.2 | TXN | Homo sapiens thioredoxin (TXN), mRNA. | 0.000 | **-1.49** |
| NM_001611.2 | ACP5 | Homo sapiens acid phosphatase 5, tartrate resistant (ACP5), mRNA. | 0.004 | **-1.56** |
| NM_002243.3 | KCNJ15 | Homo sapiens potassium inwardly-rectifying channel, subfamily J, member 15 (KCNJ15), transcript variant 2, mRNA. | 0.008 | **-1.59** |
| NM_182697.1 | UBE2H | Homo sapiens ubiquitin-conjugating enzyme E2H (UBC8 homolog, yeast) (UBE2H), transcript variant 2, mRNA. | 0.006 | **-1.63** |
| NM_016520.2 | C9orf78 | Homo sapiens chromosome 9 open reading frame 78 (C9orf78), mRNA. | 0.003 | **-1.64** |
| NM_001015880.1 | PAPSS2 | Homo sapiens 3'-phosphoadenosine 5'-phosphosulfate synthase 2 (PAPSS2), transcript variant 2, mRNA. | 0.010 | **-1.66** |
| NM_017593.3 | BMP2K | Homo sapiens BMP2 inducible kinase (BMP2K), transcript variant 2, mRNA. | 0.006 | **-1.73** |
| NM_001004023.1 | DYRK3 | Homo sapiens dual-specificity tyrosine-(Y)-phosphorylation regulated kinase 3 (DYRK3), transcript variant 2, mRNA. | 0.002 | **-1.85** |
| NM_022066.2 | UBE2O | Homo sapiens ubiquitin-conjugating enzyme E2O (UBE2O), mRNA. | 0.003 | **-1.86** |
| NM_004091.2 | E2F2 | Homo sapiens E2F transcription factor 2 (E2F2), mRNA. | 0.001 | **-1.92** |
| NM_014868.3 | RNF10 | Homo sapiens ring finger protein 10 (RNF10), mRNA. | 0.003 | **-1.93** |
| NM_020362.2 | C1orf128 | Homo sapiens chromosome 1 open reading frame 128 (C1orf128), mRNA. | 0.003 | **-2.00** |
| NM_007308.1 | SNCA | Homo sapiens synuclein, alpha (non A4 component of amyloid precursor) (SNCA), transcript variant NACP112, mRNA. | 0.004 | **-2.06** |
| NM_052828.1 | TRIM10 | Homo sapiens tripartite motif-containing 10 (TRIM10), transcript variant 2, mRNA. | 0.006 | **-2.12** |
| NM_152715.2 | LRRC35 | Homo sapiens leucine rich repeat containing 35 (LRRC35), mRNA. | 0.000 | **-2.26** |
| NM_006516.1 | SLC2A1 | Homo sapiens solute carrier family 2 (facilitated glucose transporter), member 1 (SLC2A1), mRNA. | 0.001 | **-2.27** |
| NM_016633.2 | ERAF | Homo sapiens erythroid associated factor (ERAF), mRNA. | 0.004 | **-2.28** |
| NM_000345.2 | SNCA | Homo sapiens synuclein, alpha (non A4 component of amyloid precursor) (SNCA), transcript variant NACP140, mRNA. | 0.003 | **-2.43** |
| NM_000519.3 | HBD | Homo sapiens hemoglobin, delta (HBD), mRNA. | 0.004 | **-2.47** |
| XM_931683.1 | LOC389293 | PREDICTED: Homo sapiens similar to HESB like domain containing 2, transcript variant 2 (LOC389293), mRNA. | 0.001 | **-2.49** |
| NM_005738.3 | ARL4A | Homo sapiens ADP-ribosylation factor-like 4A (ARL4A), transcript variant 1, mRNA. | 0.000 | **-2.63** |
| NM_014858.2 | TMCC2 | Homo sapiens transmembrane and coiled-coil domain family 2 (TMCC2), mRNA. | 0.000 | **-2.72** |
| NM_001010987.1 | IFIT1L | Homo sapiens interferon-induced protein with tetratricopeptide repeats 1-like (IFIT1L), mRNA. | 0.000 | **-2.76** |
| BX110640 |  | BX110640 Soares_testis_NHT Homo sapiens cDNA clone IMAGp998B094156, mRNA sequence | 0.000 | **-2.77** |
| NM_021083.2 | XK | Homo sapiens X-linked Kx blood group (McLeod syndrome) (XK), mRNA. | 0.001 | **-2.81** |
| NM_017709.3 | FAM46C | Homo sapiens family with sequence similarity 46, member C (FAM46C), mRNA. | 0.000 | **-2.92** |
| NM_003275.1 | TMOD1 | Homo sapiens tropomodulin 1 (TMOD1), mRNA. | 0.001 | **-2.96** |
| NM_199186.1 | BPGM | Homo sapiens 2,3-bisphosphoglycerate mutase (BPGM), transcript variant 2, mRNA. | 0.000 | **-3.42** |
| NM_002102.2 | GYPE | Homo sapiens glycophorin E (GYPE), transcript variant 1, mRNA. | 0.000 | **-3.84** |
| NM_002100.3 | GYPB | Homo sapiens glycophorin B (MNS blood group) (GYPB), mRNA. | 0.000 | **-4.30** |
| NM_001738.1 | CA1 | Homo sapiens carbonic anhydrase I (CA1), mRNA. | 0.000 | **-4.45** |
